# Colon biopsies: benefit or burden?

**DOI:** 10.25122/jml-2019-0009

**Published:** 2019

**Authors:** Stefan Morarasu, Muthana Haroon, Bianca Codrina Morarasu, Kirshan Lal, Emmanuel Eguare

**Affiliations:** 1. Naas General Hospital, Department of Surgery, Ireland; 2. Trinity College Dublin, Department of Surgery, Ireland; 3. Griore T Popa University of Medicine and Pharmacy, Iasi Romania; 4. Regional Oncology Institute, 2nd Clinic of Surgical Oncology, Iasi Romania

**Keywords:** Colonoscopy, diarrhea, microscopic colitis, random biopsies

## Abstract

Analyzing colon biopsies is becoming time consuming and a financial burden as colonoscopy is now the main screening and diagnostic procedure of the main gastrointestinal diseases. Colon sampling can provide important information when used accordingly; otherwise it may only load the medical system unnecessarily. Our aim was to retrospectively analyze criteria for colon biopsies and correlate the diagnostic value of randomly sampling colon, especially in patients with diarrhea. This was a retrospective study on 2109 colonoscopies done over one year. Data was collected from the ENDORAD system and included variables such as: age, gender, quality of preparation, procedure, symptoms, biopsies (type, location), and endoscopy and histology findings. Data was analyzed in a descriptive manner. Out of 496 random biopsies, only 7.4% had positive histology findings. The main symptom was diarrhea and 186 cases of patients complaining of diarrhea with normal colonoscopy had random colon sampling. In 5.3% of these cases histology assessment showed changes of microscopic colitis. Fisher’s test was significant when correlating the odds of having random biopsies in patients with and without diarrhea and patients younger and older than 60. Random sampling of colon during colonoscopies should be done only in selected patients otherwise it has a low diagnostic value.

## Introduction

Colonoscopy is the most successful diagnostic tool in the workup of a wide array of lower gastrointestinal symptoms. Moreover, it has a crucial role in colo-rectal cancer screening programs. Inflammatory bowel diseases and malignancies are the two most important pathologies that require colon tissue samples to be excluded, thus increasing the number of colonoscopies; therefore, an increasing number of biopsies are done each year building up to one of the major healthcare programs world-wide [1, 2, 3]. Given the time consumed and the financial impact sampling and analyzing biopsies has, it is important to review and establish standard guidelines on how and when to do biopsy during colonoscopies.

Diarrhea is a valid indication for colonoscopy if it is chronic (more than three watery stools per day for at least four weeks) and once infective causes have been excluded. In these cases colonoscopy is demanded to rule out inflammatory bowel disease or, in cases of normal colonic mucosa, random colon biopsies may be useful to detect microscopic colitis [4, 5, 6].

Our aim was to assess the diagnostic value of colon biopsies in the work-up of lower GI symptoms with special regards to history of diarrhea.

## Materials and Methods

This study was approved by the Research Ethics Committee of Naas General Hospital, Ireland.

We conducted a retrospective study on all colonoscopies done at Naas General Hospital over one year (2017/2018). Data was collected from the ENDORAD system and included age, gender, operator, quality of preparation, type of colonoscopy performed, symptoms, polyps (excised or not), biopsies (done or not, type of biopsy, location), endoscopic findings, and histology findings. Data were anonymous.

All data was gathered in an Excel database. Descriptive statistical analysis was done in SPSS 22.0 with the following aimed correlations:

• relationship between age, gender, and number of biopsies
• predictive diagnostic value of random biopsies in correlation with complaints of diarrhea and normal colonoscopy

## Results

Over the studied period, 2109 colonoscopies were done. Out of these, 826 procedures were done with screening purpose, while 551 were done based on clear symptomatic complaints. In the rest of the 732 cases, the indication was not reported by the operator. The cecum was reached in 1476 (70%) of cases. The terminal ileum was intubated in 421 (20%) patients, thus adding to a cecal intubation rate of 1897 (88%). The remainder were sigmoidoscopies. The preparation was noted as excellent or adequate in 1855 (87%) of cases, the rest being done under poor preparation. Regarding gender there was a slight female predominance 1159 (55%). 1265 (60%) of patients were below 60 years old, whereas the rest were older than 60.

**Figure 1:**
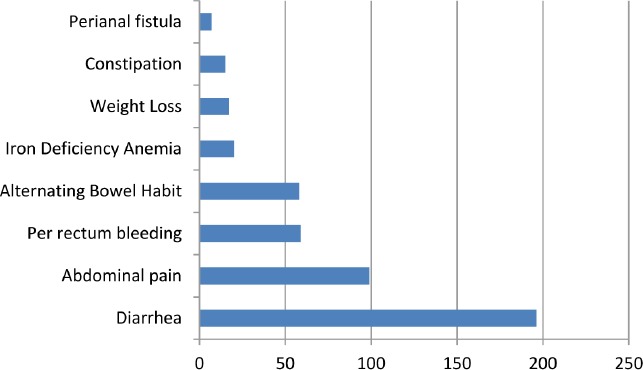
Presenting complaints of patients prior to colonoscopy out of a total of 551 patients with reported pre-procedural complaints. Patients presenting for follow-up of IBD, history of polyps or history of surgery were excluded from this graph. In this graph are included only the procedures which had a symptomatic indication, screening procedures being excluded.

The most common presenting symptom and indication for the colonoscopy was diarrhea (196 cases) followed by abdominal pain in 99 cases, per rectum bleed and alternating bowel habit (Figure 1). The majority of scopes were reported as being normal, followed by findings of polyps and colon or terminal ileum inflammation. Out of all colonoscopies, biopsies were taken in 748 (35.4%) of cases. Out of these 748 cases, 496 colonoscopies were completely normal and only random samples were taken, while in the rest, in addition, samples were taken from pathological mucosa as well.

Out of 561 polyps that were removed, 560 were benign with only one case of adenocarcinoma being described as a polyp on macroscopic appearance, the rest being either hamartoma, serrated, or low-grade dysplasia adenoma.

**Figure 2:**
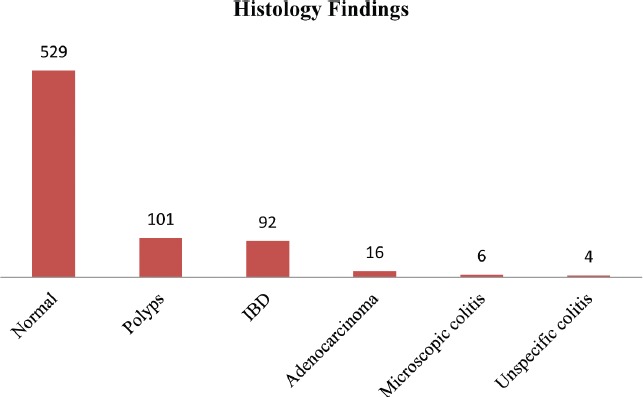
Histology findings on the sampled biopsies out of a total of 748 biopsies.

After analyzing the biopsies, the overwhelming majority were negative (530) followed by 101 reports which described polyps (all types included) and 93 with changes consistent with inflammatory bowel disease (Figure 2). Out of 496 normal colonoscopies that had biopsies taken only randomly, 37 (7.45%) had positive histology findings. In 186 cases random colonic samples were harvested to exclude microscopic changes as patients had diarrhea and normal colonoscopy. In this group of patients, 10 (5.3%) had changes consistent with microscopic colitis.

When correlating the number of biopsies with positive histology findings in a linear model, Pearson`s r was 0.15 (weak correlation). When correlating diarrhea with the probability of having random biopsies (Table 1) it was found that indeed patients complaining of diarrhea were five times (OR 5.2) more likely to have random biopsies of normal colonic mucosa, with a relative risk (RR) of 2.5 compared to patients who did not complain of diarrhea but had random colon biopsies. Fisher’s exact test was significant with p<0.00001, (CI 95%). Even so, 2.5 more patients that did not complain of diarrhea, and whose colonoscopy was normal, had biopsies harvested (404 compared to 158 which had no random biopsies taken).

**Table 1:** Contingency table correlating complaint of diarrhea and probability of random biopsies

	Random Biopsies	No random Biopsies
**Diarrhea**	173	13
**No Diarrhea**	404	158
**OR 5.3, RR 2.5, Fisher’s test p<0.00001, CI 95%**		

From a total of 186 patients which had diarrhea and normal colonoscopy, 173 had random biopsies while 13 did not have. When compared to patients which did not present with diarrhea and had (or not) biopsies, Fisher’s test p<0.00001 showing significant correlation, patients with diarrhea having significant more biopsies harvested.

With regards to age, patients younger than 60 years old were six times more likely (OR 5.7) to have random biopsies taken compared to siblings older than 60. Again, Fisher’s exact test confirmed significance with p<0.00001 (Table 2). Even when analyzing only patients younger than 60 years old with complaints of diarrhea, more random biopsies were taken in patients younger than 60, regardless of gender. Sixty-four males (81%), less than 60, with diarrhea had random biopsies compared to only 15 (19%) over 60. Fifty-six females (62%), younger than 60, with diarrhea had random colonic biopsies compared to 33 over 60 years old (38%).

**Table 2:** Contingency table correlating age with probability of having random biopsies

	Random Biopsies	No random Biopsies
**<60 years old**	426	322
**>60 years old**	140	608
**OR 5.7, RR 3.0, Fisher’s test p<0.00001, CI 95%**		

Significant correlation suggests younger patients (less than 60) are more likely to have random colonic samples than patients older than 60.

## Discussions

In patients with normal colonoscopies and random biopsies, the rate of positive findings was 7.4%, which implies a low predictive value of randomly sampling colon, and other studies [7, 8, 9]. showed comparable low rates (Table 3). To note, our cecal intubation rate was 88%, which is less than the recommended 90%, thus the results may be biased when considering this aspect.

**Table 3:** Rate of positive histology findings in patients randomly biopsied with normal colonoscopies (literature comparison)

	Our study	Genta et al.^1^	Elliot et al.^2^
Result	7.4 %	19 %	9.7 %

Microscopic colitis, with its two subtypes lymphocytic and collagenous colitis is a potential cause of watery diarrhea, especially in elderly female patients. In our study, more random biopsies to exclude microscopic colitis were sampled in females, however significantly more in patients younger than 60-years-old. This emphasizes the need to correctly select patients that require random colon sampling.

The most common symptomatic complaint was diarrhea. Random colon biopsies are recommended in patients with diarrhea to exclude microscopic colitis. However, only patients with chronic diarrhea should be guided towards colonoscopy and colon sampling and only after infective causes have been excluded. Herein there is a bias regarding the nature of diarrhea as there was not a selection of patients that only had symptoms consistent with the definition of chronic diarrhea: at least three loose stools per day for at least four consecutive weeks. This bias would partially explain the low predictive rate of only 5.3% when compared to other studies [7, 9, 10] which analyzed only patients with chronic diarrhea (Table 4). This difference in rates emphasizes the need to randomly sample the colon only in selected patients which have chronic diarrhea and no infectious causes [11, 12].

**Table 4:** Rate of microscopic colitis in patients with random colon biopsies for diarrhea (literature comparison)

	Our study	Kagueyama et al.^3^	Hotouras A et al.^4^	Genta RM et al.^1^
Result	5.3%	49.45%	1.3 %	8.6 %

The idea of not removing polyps or at least not sending them for histologic analysis is indeed an unnecessary pressure for the endoscopist given the fact that there is a small risk one of the discarded ones could be an adenocarcinoma in situ. On the other hand, sending all polyps for histological examination is a financial burden and most time-consuming. In our study, 561 polyps were removed and 560 of them were benign (serrated polyp, hamartoma, or low-grade dysplasia adenoma). With the advent of narrow banding imaging the macroscopic analysis of polyps *in situ* is more specific and this could be a useful objective tool in deciding which polyps can be safely discarded [13].

## Conclusions

Colon biopsies have increased diagnostic value when sampled in selected patients. Random colon sampling in patients with diarrhea may be useless if not used based on specific criteria.

## Conflict of Interest

The authors confirm that there are no conflicts of interest.
